# Potential Adverse Effects of Urinary Bisphenol A Concentrations on Renal Function, Gestation, and Newborn Characteristics in Healthy Pregnant Women

**DOI:** 10.3390/jox16060180

**Published:** 2026-09-24

**Authors:** Senka Imamović Kumalić, Gorazd Kavšek, Mateja Sladič, Tanja Premru-Sršen, Irma Virant-Klun

**Affiliations:** 1 Division of Obstetrics and Gynecology, Department of Perinatology, University Medical Centre Ljubljana, Šlajmerjeva 3, 1000 Ljubljana, Slovenia; 2 Medical Faculty, University of Ljubljana, 1000 Ljubljana, Slovenia; 3 Clinical Research Centre, University Medical Centre Ljubljana, Vrazov Trg 1, 1000 Ljubljana, Slovenia

**Keywords:** endocrine-disrupting chemical, bisphenol A, urine, pregnant women, newborn

## Abstract

The impact of bisphenols on the metabolism and kidney function of pregnant women, and consequently on the health of the child, is not yet well known. This study investigated urinary concentrations of bisphenols (BPA, BPF, and BPS) and their associations with biochemical parameters for kidney and liver function, serum anti-Müllerian hormone (AMH), vitamin D3, and pregnancy outcomes in pregnant women. Fasting morning urine and blood samples were collected from 80 healthy women with singleton, third-trimester pregnancy to quantify urinary BPA, BPF, and BPS concentrations and analyze routine biochemical parameters for kidney and liver function. BPA was detected in 78% of urine samples, compared with 10% for BPF and 5% for BPS, making it the predominant bisphenol. BPA concentrations positively correlated with serum creatinine, and this relationship remained significant after adjustment for maternal age, body mass index, gestational age, and urine specific gravity in multivariable linear regression analysis. Its concentrations were also positively correlated with AMH and negatively with gestational duration and newborn weight and head circumference, although these associations were not confirmed by linear regression analyses. These findings suggest that BPA exposure during late pregnancy may influence maternal kidney function and potentially ovarian reserve, as well as neonatal outcomes, warranting further investigation.

## 1. Introduction

Many studies have reported that endocrine-disrupting chemicals (EDCs) can affect reproduction and the development of exposed humans [1,2,3,4,5]. Once inside the body, EDCs primarily bind to nuclear estrogen receptors (ERα and ERβ) or membrane receptors (GPER, ER-X) in different tissues [6], acting as false “estrogens”, a mechanism that has been linked to infertility and other reproductive disorders in women [7,8,9,10,11,12].

In most cases, humans are exposed to EDCs through the ingestion of contaminated food, with bisphenol A (BPA), nonylphenol, phthalates, and heavy metals being the most common [4,5].

An important group of EDCs is represented by bisphenols (BPs). BPA is one of the most produced toxic chemicals worldwide, the most well known, and perhaps the most studied bisphenol along with its analogs bisphenol F (BPF) and bisphenol S (BPS). It is widely used as a key monomer of epoxy resins and polycarbonate (PC) plastics to improve their quality [13,14,15] and is released from plastic products used to store and warm up food and drink, thermal invoice paper, and components of children’s products such as baby bottles and toys [16]. 

BPA has been largely studied as an obesogenic agent that might contribute to weight gain, insulin resistance, and pancreatic β-cell dysfunction in pregnancy, potentially playing a role in the development of pregnancy complications, such as gestational diabetes mellitus (GDM), and adverse outcomes [17]. However, a systemic review and meta-analysis showed no association between exposure to BPA during pregnancy and the risk of GDM/impaired glucose tolerance [18]. On the other hand, BPA exposure has been linked to certain placenta-associated obstetric complications such as preeclampsia, fetal growth restriction, miscarriage, and preterm birth; however, the mechanisms resulting in these disorders remain uncertain [19]. Much less is known about the impact of BPA on the metabolism of pregnant women in terms of kidney and liver function and newborn characteristics. BPA exposure during pregnancy might negatively affect both maternal and fetal kidney health by inducing oxidative stress, inflammation, and abnormal renal development, although there is not much experimental evidence to date in humans [20]. Moreover, some recent studies highlighted a relationship between maternal exposure to EDCs including BPA, development of the embryo, feto-placental growth, and fetal outcome and negative consequences for the health of offspring, even later in life [21].

Pregnant women are regularly exposed to a variety of environmental toxicants in their daily life, posing a potential risk of kidney injury before the presence of clinical manifestations and signs. Because the impact of bisphenols on the metabolism of pregnant women, and consequently on the health of the child, is not yet well known, the aim of this study was to determine the content of bisphenols—BPA, BPF, and BPS—in the urine of healthy pregnant women and investigate any possible influence of the most abundant BPA on maternal urine and blood biochemical parameters related to kidney and liver function, fat metabolism, AMH, and vitamin D3 and characteristics of delivery and the newborn.

## 2. Materials and Methods

### 2.1. Study Design and Population

The observational study was performed from September 2021 to October 2024 at the Department of Obstetrics, Division of Obstetrics and Gynecology, of the University Medical Centre Ljubljana, Slovenia. It was carried out within the framework of the research project J3-2530 and research program P3-0124, which were supported by the Public Agency for Scientific Research and Innovation (ARIS) and approved by the Slovenian National Medical Ethics Committee (consent number 0120-448/2020-3, from 5 November 2020). All participants were enrolled after they provided written informed consent to participate in the study; each participant received a code to pseudonymize the results, and all samples and results were managed and analyzed under this code.

#### Participants

A total of 80 women with a singleton pregnancy between 37 and 41 weeks of gestation (third trimester), aged 20 to 38 years, were enrolled after they signed a written informed consent form to participate in this study. Samples of blood and urine were taken after fasting for at least 6 h. Due to limited criteria, it was easiest to include those admitted for planned cesarean section at term, mostly due to previous cesarean section. The exclusion criteria were an age of 38 years or more; twin or multiple pregnancy; chronic diseases such as diabetes, Crohn’s disease, asthma, diseases of the musculoskeletal system, celiac disease, chronic obstructive pulmonary disease, and cardiovascular, liver, kidney, neurological, thyroid (hypothyroidism was allowed), and oncological diseases; and complications during pregnancy such as gestational hypertension or preeclampsia, GDM treated with insulin, fetal growth restriction, and termination of pregnancy due to fetal malformations or fetal demise.

### 2.2. Sampling Procedure

One urine and one blood sample were collected from each woman. Within a maximum of two hours, all urine and blood samples were divided into 1.5 mL aliquots using an automated pipette. Fresh samples of urine and blood (serum) were biochemically analyzed with usual diagnostic laboratory tests in daily clinical practice, mainly for liver and kidney function, fat metabolism, and vitamin D3, and the rest of the urine samples were deep-frozen and stored at −80 °C for subsequent analysis of bisphenols A, F, and S (BPA, BPF, and BPS). Once all samples were collected, they were thawed and simultaneously analyzed for BPA, BPF, and BPS. Remaining blood samples (serum) were analyzed for anti-Müllerian hormone (AMH).

### 2.3. Diagnostic Biochemical Analyses of Blood and Urine

Urine and serum samples of women were analyzed at the Clinical Institute for Clinical Chemistry and Biochemistry (KIKKB) at the University Medical Center Ljubljana with various established and validated methods that are used in daily diagnostics in certified medical laboratories. The women’s urine was analyzed for the following parameters, particularly to determine kidney function: specific density, IgG/creatinine, albumin/creatinine, alpha-1-microglobulin/creatinine, IgG/U-albumin, creatinine, N-acetyl-β-d-glucosaminidase (NAG)/creatinine, and NAG.

The women’s serum samples were analyzed for the following to determine hemogram, fat metabolism, and kidney and liver function: creatinine, oGF (glomelural filtration estimation), AST (aspartate aminotransferase), ALT (alanine transaminase), gamma-GT (gamma-glutamyl transferase), cholesterol, HDL cholesterol, LDL cholesterol, TG (triglycerides), WBC (white blood cell count), RBC (red blood cell count), Hb (hemoglobin), Ht (hematocrit), MCV (mean erythrocyte volume), MCH (mean erythrocyte hemoglobin content), MCHC (average erythrocyte concentration of hemoglobin), RDW (volume distribution of erythrocytes), K-thrombocytes/platelets (their count), MPV (average volume of thrombocytes/platelets), and 25-hydroxy vitamin D (vitamin D3).

Total 25-hydroxyvitamin D concentration in serum was quantitatively determined using the Abbott automated chemiluminescent microparticle immunoassay (CMIA) method.

### 2.4. AMH Analysis

Analysis was performed at the Medicare PLUS diagnostic lab in Ljubljana. The concentration of AMH in serum samples was determined by the electrochemiluminescence immunoassay (ECLIA), using the sandwich principle, on the Roche Cobas e411 apparatus. During the first incubation, 50 µL of sample, biotin-labeled AMH-specific monoclonal antibodies and ruthenium complex-labeled AMH-specific monoclonal antibodies reacted with each other to form a sandwich complex. During the second incubation, streptavidin-coated microparticles were added. The complex bound to the solid phase due to the interaction between biotin and streptavidin; then, the reaction mixture traveled to the measuring cell, and microparticles were trapped on the surface of the electrode due to the magnetic action. Unbound materials were removed by ProCell M. Voltage across the electrode induced chemiluminescence emission, which was then measured by a photomultiplier. The analyzer then recalculated the concentration with the help of a calibration curve, which was obtained using a 2-point calibration. Concentrations of AMH were expressed in ng/mL.

### 2.5. Analyses of Bisphenols—BPA, BPF, and BPS

Urine samples were analyzed for bisphenols at the Department of Environmental Sciences, Jožef Stefan Institute, Ljubljana, according to the established methodology [22]. The samples were deconjugated with the enzyme β-glucoronidase/arylsulfatase and then extracted on the solid phase. After elution from the polymer sorbent, they were dried, reconstituted in acetonitrile, derivatized, and then purified on SiOH plates, concentrated in a stream of nitrogen to approximately 100 µL, and analyzed by gas chromatography–tandem mass spectrometry (GC-MS/MS). After this, the samples were analyzed by two different methods: with the first method, BPA and BPF were determined; then, more derivatizing agent was added to the samples and BPS was determined. The measurements above the defined “Limit of Detection” (LOD) for each bisphenol, that is, the lowest concentration in a sample that can be consistently detected with a certain level of certainty, typically 95%, were respected.

### 2.6. Mother, Delivery, and Newborn Data

Data on mothers (age, region of residence, body mass index—BMI, parity, number of previous pregnancies and spontaneous abortions), pregnancy (GDM on diet, hypothyroidism), labor, and newborns (Apgar after 5 and 10 min, pH arterial, pH venous, gender, weight, length, head circumference) were collected from the same women to relate them to BPA concentrations in their urine. GDM screening was performed according to the applicable Slovenian professional guidelines and national recommendations [23].

### 2.7. Statistical Analyses

Statistical analyses were performed using urinary BPA concentrations as non-adjusted (µg/L), creatinine-adjusted (µg/g creatinine), or urine specific gravity-adjusted data. The Shapiro–Wilk test was used to determine the distribution of the data (results); parametric (One-Way ANOVA) and non-parametric statistical tests (Kruskal–Wallis test, Spearman’s correlation coefficient, Fisher’s exact test) were used for the analysis of normally and abnormally distributed data, respectively. The relationship between the contents of BPA in the urine, the results of diagnostic biochemical tests, AMH, vitamin D3, and characteristics of the newborn was determined using Spearman’s correlation test. Associations between BPA concentrations and continuous maternal urinary and blood (serum) biochemical parameters, as well as obstetric and neonatal outcomes, were evaluated using multivariable linear regression models. Associations with binary outcomes were examined using multivariable logistic regression models. Covariate selection was informed by the prior literature [24,25,26,27,28,29,30,31,32,33,34,35,36] and biological plausibility. Spearman correlation coefficients were examined to screen for multicollinearity, and covariates with |ρ| < 0.6 were retained for joint inclusion in regression models. All statistical analyses were performed using IBM SPSS Statistics, version 29 (IBM Corp., Chicago, IL, USA), and statistical significance was defined as a two-sided *p*-value < 0.05.

### 2.8. Missing Data

The study cohort consisted of 80 participants; the biochemical analysis was unsuccessful for three of them, resulting in 77 participants with available biochemical and urinary BPA measurements. Additional missing data were due to incomplete self-administered questionnaires, as some participants did not provide responses to all questionnaire items. No participants were excluded after enrollment because of missing questionnaire data. Analyses were performed using the available observations for each variable, and the corresponding sample size (*n*) is reported where applicable.

## 3. Results

### 3.1. Study Population Characteristics

The characteristics of the study population of pregnant women and their neonates are summarized in Table 1 and Table 2, with additional details provided in Supplementary Materials Tables S1–S3. Participants in the study cohort had a median age of 33 years (range: 23–39) and a median BMI of 24.1 kg/m^2^ (range: 18.2–40.7), and the majority were multiparous (55%), tertiary-educated (57.6%), and non-smokers (96.7%), with 95% reporting spontaneous conception. Approximately 77.5% of women resided in the Central Slovenian Region, and diet-controlled GDM was reported by 28.7% of participants.

The neonates of these women were delivered at a median gestational age of 39 weeks with equal sex distribution. Median birth weight, length, and head circumference were 3520 g, 51 cm, and 35 cm, respectively; median Apgar scores were 9 at both 1 and 5 min, and median umbilical arterial and venous pH values were 7.30 and 7.35, as shown in Table 2.

### 3.2. Concentrations of Bisphenols in Urine of Pregnant Women

The LOQ (limit of quantification) and LOD values were 0.10/0.03 μg/L, 0.10/0.03 μg/L, and 0.50/0.15 μg/L for BPA, BPF, and BPS, respectively.

The mean (average), median, and geometric mean values for non-adjusted BPA concentration in urine were 0.2813 ± 0.1841 μg/L, 0.220 μg/L, and 0.2374 (0.101–1.04) μg/L, respectively. After normalization for creatinine, these values for BPA were 0.4339 ± 0.1184 μg/g creatinine, 0.3526 μg/g creatinine, and 0.3529 (0.0748–1.7433) μg/g creatinine, respectively. Geometric means and geometric standard deviations of urinary bisphenol concentrations are shown in Supplementary Materials Table S4.

BPA was detected in 78% (60 out of 77) of samples (women). The frequency of BPA concentrations in urine samples can be seen in Figure 1: the majority have concentrations of BPA up to 0.400 μg/L, with the highest peak between 0.100 and 0.200 μg/L. In this range, normal values of BPA were expected in urine samples of pregnant women based on observed values (Figure 2). In a proportion of samples, namely eight (10%), BPA concentrations exceeded these values, even 1.000 μg/L, and were expected to be abnormal.

Among other bisphenols, BPF was detected in 10% (8 out of 77) and BPS in 5% (4 out of 77) of urine samples (Figure 3). Since BPA was by far the most abundant bisphenol in the urine of pregnant women, it was further correlated to the characteristics of these women and their newborns. BPF concentrations (*n* = 8, range: 0.102–0.369 μg/L; mean (average) value: 0.2015 ± 0.0865 μg/L, median value: 0.1945 μg/L, geometric mean: 0.1842 μg/L) tended to be lower than those of BPA, while BPS was expressed in a smaller proportion of urine samples (5%) but in higher concentrations (*n* = 4, range: 0.334–8.440 μg/L; mean (average) value: 4.1307 ± 3.6454 μg/L, median value: 3.8745 μg/L, geometric mean value: 1.9368 μg/L) than the other two bisphenols, as shown in Figure 3.

### 3.3. BPA in Maternal Urine and Female Characteristics

Our data show that urinary levels of BPA adjusted for creatinine in women were not at all significantly related to characteristics such as region of residence (r = 0.0012), way of conception (r = 0.0122), BMI (r = −0.1191), parity (r = 0.11), number of previous pregnancies (r = 0.1893) and spontaneous abortions (r = 0.1893), GDM on diet (r = −0.0461), and hypothyroidism (r = −0.0349) (Table 3).

### 3.4. BPA in Maternal Urine and Neonatal Characteristics

Among neonatal characteristics, creatinine-adjusted maternal urinary BPA concentrations were negatively correlated with gestational age at birth (r = −0.3494), newborn birth weight (r = −0.264), and head circumference at birth (r = −0.301). The correlations with newborn birth weight and head circumference were based on raw neonatal measurements and were obtained from univariate Spearman analyses without adjustment for gestational age at birth. No correlations were observed with neonatal sex, Apgar score at 1 min, pH-A, or pH-V (Table 4). In the subsequent multivariable linear regression analyses, gestational age at birth was included as a covariate, and the associations with newborn birth weight and head circumference were not confirmed.

### 3.5. Correlations Between BPA and Maternal Urine and Biochemical Parameters Related to Kidney and Liver Function, AMH, and Vitamin D3

Urinary BPA concentrations adjusted for creatinine were negatively correlated with urine specific gravity (r = −0.546) and serum triglycerides (r = −0.376), as shown in Table 5. There was also a significant positive correlation of urinary BPA with serum AMH concentrations (r = 0.298).

### 3.6. Multivariable Linear Regression Analysis of the Association Between Urinary BPA Concentrations and Other Maternal and Neonatal Characteristics

This analysis did not show any significant associations between urinary BPA concentrations adjusted for creatinine and female or neonatal characteristics. However, a consistent positive association was observed between urinary BPA concentrations and maternal serum creatinine levels in pregnant women (Figure 4).

Models were adjusted for BMI, maternal age, and gestational age, with additional adjustment for GDM, parity, or education. Urine dilution was accounted for using either urine specific gravity (SG) or urinary creatinine (Table 6).

### 3.7. Multivariable Linear Regression Analysis of the Association Between Urinary BPA Concentrations and Maternal as Well as Female Neonate Characteristics

Among pregnancies resulting in female neonates, the regression model for maternal serum creatinine was not statistically significant (*p* = 0.336), and the association between ln-transformed BPA adjusted for urine specific gravity and serum creatinine did not reach statistical significance (B = 13.49, 95% CI: −3.06 to 30.04, *p* = 0.105), although the direction and magnitude of the effect were comparable (Table 7). The analysis showed significant associations between urinary BPA concentrations adjusted for specific gravity and maternal platelet value (*p* = 0.02).

### 3.8. Multivariable Linear Regression Analysis of the Association Between Urinary BPA Concentrations and Maternal as Well as Male Neonate Characteristics

Among pregnancies resulting in male neonates, the regression model for maternal serum creatinine was statistically significant (*p* = 0.049), explaining 37.8% of its variance. In this subgroup, higher ln-transformed urinary BPA concentrations were associated with higher serum creatinine (B = 21.13, 95% CI: 4.62–37.63, *p* = 0.014), corresponding to an approximate increase of 2.0 µmol/L per 10% increase in BPA (Table 8). The analysis showed significant associations between urinary BPA concentrations adjusted for specific gravity and maternal MPV (*p* = 0.034).

Although these subgroup-specific results may suggest a stronger association in the male subgroup, the interaction between BPA and newborn gender, assessed by including an interaction term in the primary regression model of the overall cohort, was not statistically significant (*p* = 0.470), indicating no statistical evidence that the association differs by gender in the observed cohort of newborns.

## 4. Discussion

The impact of EDCs, specifically bisphenols, on the metabolism of pregnant women, and consequently on the health of the child, is not yet well known; therefore, the aim of this study was to determine the content of BPA, BPF, and BPS in urine samples of pregnant women and their relation to maternal and neonatal characteristics. It was found that BPA was by far the most prevalent bisphenol in urine compared with its analogs BPF and BPS, which were detected only sporadically. There was a consistent positive correlation between urinary BPA and serum creatinine in pregnant women, confirmed by multivariable linear regression, thus indicating possible kidney damage. Moreover, there were also some correlations between urinary BPA concentrations and serum AMH, gestation, and some newborn characteristics such as weight and head circumference; however, these findings need to be explored further.

The levels of urinary BPA found in this study are relatively low in comparison to those in other studies in pregnant women worldwide (Supplementary Materials Table S5). Similarly to our study (78%), BPA was detected in the majority of urine samples: 100% in China [37]; 71.5% in Japan [38]; 93.3% in Oklahoma, USA [39]; 100% on the Caribbean islands [40]; 59% in California, USA [41]; and 76% in Spain [42]. The median BPA concentration in this study of 0.22 µg/L (third trimester) is lower than that in other studies: 5.84 μg/L (third trimester) in China [37,43], 1.29 μg/L (third trimester) in Denmark [44], and 0.81 µg/L in Canada [45]. Similarly, the median BPA concentration adjusted per g of creatinine in this study (0.3526 μg/g creatinine) is lower than that in other studies: 0.46 μg/g creatinine in Japan [38], 1.58 μg/g creatinine (third trimester) in Turkey [46], and 2.51 μg/g creatinine (third trimester) in Spain [42]. This indicates a relatively low exposure of Slovenian pregnant women to BPA in comparison to some other studies from different countries.

This is the first study on BPA in pregnant women in Slovenia. There is only one related study in the Slovenian general population, which showed that the urinary levels of total BPA in children and their mothers and fathers were relatively low, with geometric means of 1.51, 0.79, and 0.20 μg/g creatinine, respectively; these are comparable to the levels reported for other European countries, below the current health-based guidance values [47,48], and higher than the levels in pregnant women in this study. These findings suggest that, despite regulatory efforts, BPA exposure during pregnancy remains a common problem. Importantly, the relatively low BPA concentrations in pregnant women observed in our cohort provide an opportunity to explore whether even low-level exposure may be associated with subtle metabolic or developmental changes in pregnant women and their newborns.

Creatinine-adjusted BPA concentrations in urine were positively correlated and associated with serum concentrations of creatinine, as revealed by multivariable linear regression models adjusted for BMI, maternal age, and gestational age, with additional adjustment for GDM, parity, or education, thus indicating the potential impairment of kidney function. An important methodological consideration is the potential mathematical and physiological coupling associated with creatinine normalization of urinary BPA when serum creatinine is evaluated as an outcome. Because urinary and serum creatinine are both related to renal function, creatinine-adjusted urinary BPA concentrations may potentially contribute to an apparent association with serum creatinine independent of a direct biological effect of BPA on kidney function. However, in our analysis, the association between BPA and serum creatinine remained statistically significant when urinary BPA concentrations were adjusted for specific gravity rather than urinary creatinine, suggesting that the observed association is not solely attributable to urinary creatinine normalization. Nevertheless, this finding should be interpreted cautiously, and the possibility of residual physiological confounding cannot be completely excluded. In sex-stratified analyses, the association between BPA and serum creatinine was statistically significant only in pregnancies with male newborns. However, the interaction test was not statistically significant, indicating no strong evidence for effect modification by sex; therefore, this finding should be interpreted with caution and may reflect limited statistical power rather than true biological differences. Several recent studies have shown a link between BPA and kidney damage in animal models such as mice [49,50,51] and rats [52], finding that BPA disrupts renal metabolism via MAPK-mediated steroid hormone dysregulation identified by transcriptomic and metabolomic analyses [50]. Chen et al.’s study (2025) in mice also showed that estrogen receptor alpha (ERα) serves as a critical molecular nexus linking environmental BPA exposure to macrophage apoptosis-driven renal pathophysiology [52]. BPA may also negatively affect the kidneys by increasing oxidative stress in animals: rats that were exposed to BPA showed a decrease in antioxidant glutathione (GSH), serum sodium, and potassium levels, and an increase in the level of a key biomarker of oxidative stress, malondialdehyde (MDA), produced as a byproduct of lipid peroxidation (oxidative degradation of lipids) [52]. These biochemical results were also supported by histopathological changes in renal tissues. Pre-treatment with antioxidant vitamin C in animals showed a rise in GSH, sodium, and potassium levels and a fall in MDA, as well as some beneficial changes in kidney histology after BPA exposure. Human studies are rare. However, a sensitive risk factor for abnormal NAG/creatinine levels after exposure to BPA and its substitutes was found in a Taiwanese general population [53]; in this study, urinary BPA and exposure to its substitutes increased the risk of renal tubular injury (N-acetyl- *β*-d-glucosaminidase).

In this study, our observations in pregnant women are further supported by a significant negative correlation between creatinine-adjusted urinary BPA concentrations and urine specific gravity, suggesting a possible interaction between BPA exposure and renal handling of water and solutes. However, our data need to be interpreted with caution because the kidney physiology may be affected by pregnancy itself. It is clear that potential physiological changes during pregnancy must be considered. For example, significant volume expansion and vasodilation in systemic and kidney hemodynamics can be observed. Moreover, a series of studies suggest an overall progressive increase in glomerular filtration rate (GFR) approximating 40% to 50%, with peak increases sustained at term in uncomplicated pregnancies [54,55], which may lead to lower urine specific gravity. Renal plasma flow (RPF) in pregnant women also increases up to 80% compared with “non-pregnant” levels [56]. Furthermore, tubular function and handling of water and electrolytes in kidneys are altered, leading to a mild increase in proteinuria and glucosuria, lower serum osmolality, and reductions in serum sodium levels [57]. During a normal pregnancy, increased blood volume and kidney function also cause an increase in the amount of creatinine filtered out of the blood and passed into the urine. However, the strengths of this study include the well-characterized cohort of healthy pregnant women with singleton pregnancy only and in the same third trimester of pregnancy (just before a planned cesarean section at term). An important question arises as to how impaired kidney function in pregnant women due to BPA exposure may affect the health of the child.

Our research also found other interesting correlations with BPA in urine of pregnant women that are worth mentioning; however, since they were not confirmed by regression analysis, they need to be investigated further. There was a significant negative correlation between urinary BPA concentrations and serum triglyceride levels, which may be related to liver function. This finding aligns with some epidemiological studies reporting inverse associations between BPA exposure and serum triglycerides in humans. For example, a Chinese study in around 1870 adults aged over 40 years showed that doubling urinary BPA was associated with higher LDL, non-HDL, and TC/HDL ratio but lower triglyceride levels in repeated measures analysis [58]. Another study showed that European pregnant women showed lower second- and third-trimester triglycerides compared with those from Asia [59]. The observed inverse association between urinary BPA and serum triglycerides in the third trimester of pregnancy may reflect the complex interactions between endocrine disruption and pregnancy-specific metabolic adaptations rather than a direct causal effect. We observed statistically significant associations between urinary BPA and platelet-related parameters, including platelet count and MPV, in some regression analyses; however, the overall models were not statistically significant and the findings were not consistent across the different models, so these results should be interpreted with caution. Limited evidence is available regarding the potential effects of BPA on platelet-related parameters; experimental studies have suggested that it may influence platelet activation and aggregation, potentially through oxidative stress-related mechanisms [60].

Our study identified significant negative correlations between maternal urinary BPA concentrations and gestational duration, newborn birth weight, and head circumference. However, these associations were observed in unadjusted correlation analyses and were not confirmed in multivariable linear regression analyses; therefore, they should be interpreted with caution and should not be considered evidence of an independent association between maternal BPA exposure and these pregnancy or neonatal outcomes. Nevertheless, the observed correlations are of potential interest and warrant further investigation. Some studies have suggested that maternal urinary BPA exposure may impair fetal growth [61,62], potentially through placental dysfunction, altered nutrient transport, or endocrine-mediated mechanisms. In contrast, a published meta-analysis found that maternal urinary BPA concentrations were positively associated with birth weight but not with birth length, head circumference, or gestational age at birth [63]. These inconsistent findings across studies may reflect differences in study populations, timing and levels of BPA exposure, exposure assessment, and adjustment for potential confounding factors. Further research in larger prospective cohorts is needed to clarify whether maternal BPA exposure is associated with pregnancy duration and neonatal growth and to determine the potential clinical relevance of these observations.

An additional finding of this study was the small but significant positive correlation between urinary BPA concentrations and serum AMH levels, although this was not confirmed by multivariable regression analysis. This observation contrasts with some other studies reporting negative or null associations between BPA exposure and AMH, particularly in non-pregnant women or women with infertility [64,65]. It is also important to consider the physiological changes in serum AMH concentrations during pregnancy: Köninger et al. investigated AMH concentrations during pregnancy and reported significant differences between trimesters, with concentrations declining during pregnancy and increasing during the first four days postpartum [66]. Therefore, AMH levels assessed during pregnancy may not accurately reflect ovarian reserve, as concentrations appear to be influenced by gestational age [66]. A possible biological explanation for the unexpected positive association observed in our study is that BPA may interfere with the regulation of the AMH pathway. Experimental evidence in bovine granulosa cells has shown that BPA exposure alters downstream components of the AMH/AMHRII-SMAD signaling pathway, suggesting that BPA may interact with cellular mechanisms involved in AMH signaling [67]. In addition, BPA has been shown to interfere with ovarian endocrine signaling and steroidogenesis through several receptor- and enzyme-mediated pathways [68]. These findings provide a plausible biological basis for an association between BPA exposure and AMH regulation, although they do not establish that BPA increases circulating AMH concentrations in humans. Importantly, these experimental findings cannot be directly extrapolated to healthy pregnant women. The mechanism underlying the positive association observed in our study therefore remains uncertain and should be considered hypothesis-generating. Furthermore, experimental evidence suggests that BPA may affect reproductive processes beyond ovarian endocrine signaling, as BPA exposure has been reported to impair oocyte maturation and subsequent embryo development in an experimental porcine model [69]. Consequently, the positive association observed in our study should not be interpreted as evidence of a beneficial effect of BPA on ovarian reserve, but rather as an association that requires further investigation. Further research is needed to confirm these findings and clarify the underlying mechanisms. A further methodological consideration is that AMH was analyzed at an external laboratory using an electrochemiluminescence immunoassay (ECLIA) on a Roche Cobas e411 analyzer, whereas the routine biochemical parameters were analyzed at our institution using a different analytical platform. Although validated laboratory methods were used, potential inter-laboratory and inter-assay variability should be considered when interpreting the observed association between BPA and AMH.

An important limitation of this study is the assessment of BPA exposure using a single-spot urine sample. Due to the relatively short biological half-life of BPA, urinary concentrations may vary considerably over time and primarily reflect recent exposure rather than usual or long-term exposure. Therefore, a single urine measurement may not adequately characterize cumulative or chronic BPA exposure and may result in exposure misclassification. This limitation should be considered when interpreting the observed associations between urinary BPA concentrations and maternal and neonatal outcomes.

Further research is needed to draw definitive conclusions. In the future, studies should include a larger number of pregnant women, preferably in multicenter settings, and use repeated urinary BPA measurements at different time points during pregnancy to better characterize maternal BPA exposure.

## 5. Conclusions

In conclusion, BPA was the predominant bisphenol detected in the urine of healthy women in the third trimester of singleton pregnancy, occurring at levels that are relatively low in comparison to pregnant women in other studies worldwide. Even at these relatively low concentrations, maternal urinary BPA was associated with alterations in renal-related urinary parameters such as serum creatinine, as confirmed by multivariable linear regression. Although the association appeared stronger in pregnancies with male rather than female newborns, this difference between genders was not statistically confirmed. There were also some correlations between urinary BPA and serum AMH, gestation, and selected neonatal anthropometric outcomes such as birth weight and head circumference, although these were not confirmed by multivariable linear regression analysis. While the interpretation of maternal biochemical markers during pregnancy is inherently complex due to several physiological adaptations, the observed potential associations with newborn weight and head circumference raise concern about possible effects of prenatal BPA exposure on fetal growth but need to be further investigated in a higher number of pregnant women. These observations support growing evidence that even low-dose exposure to endocrine-disrupting chemicals including BPA during pregnancy may have biologically relevant effects. Future studies need to be performed, especially longitudinal exposure assessments across pregnancy, incorporate mixtures of endocrine disruptors, and examine long-term child health outcomes to better understand the clinical relevance of these associations.

## Figures and Tables

**Figure 1:**
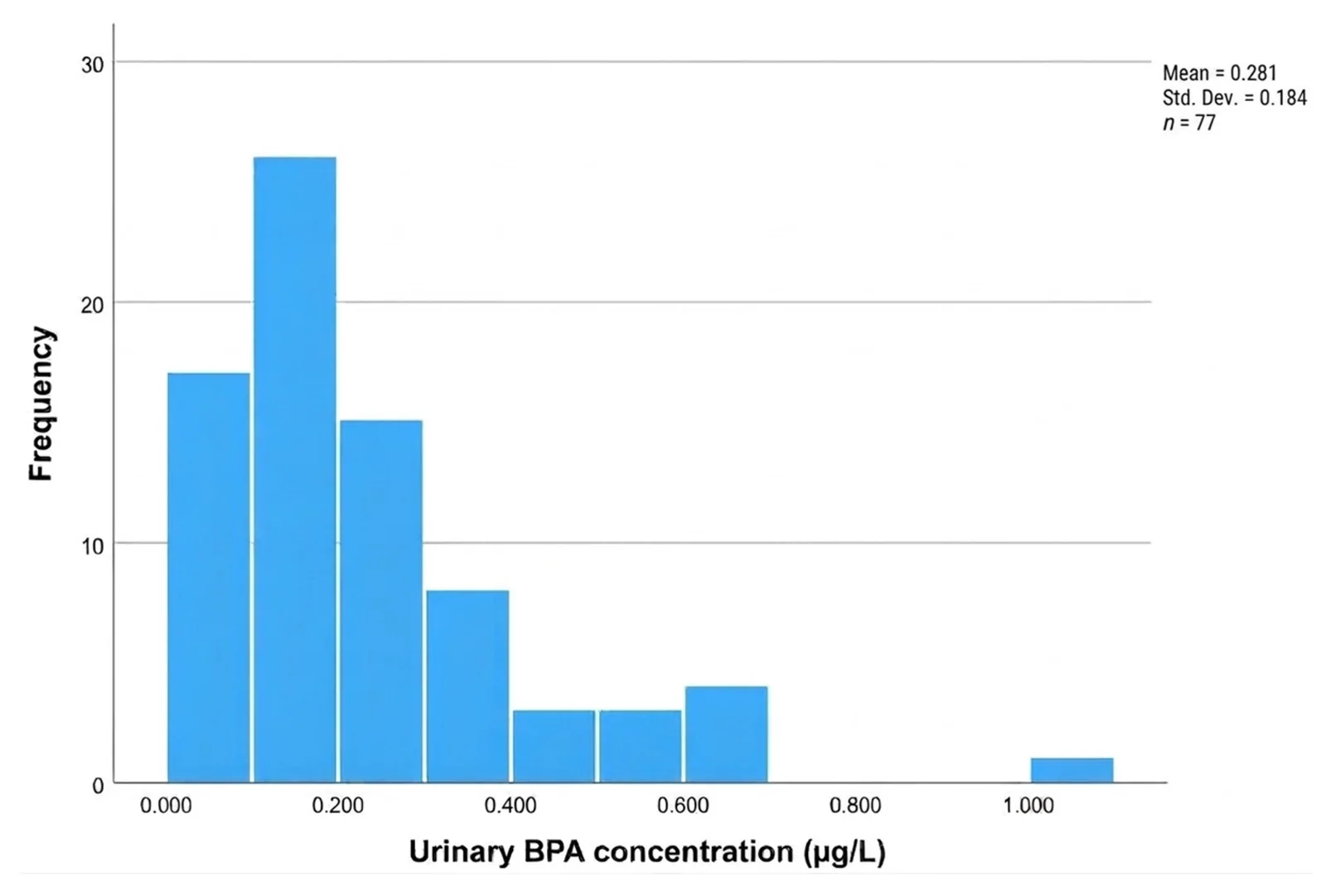
Frequency of BPA concentrations in urine samples of 77 pregnant women (third trimester). In the majority of samples, the concentrations of BPA were up to 0.400 μg/L, with the highest peak between 0.100 and 0.200 μg/L.

**Figure 2:**
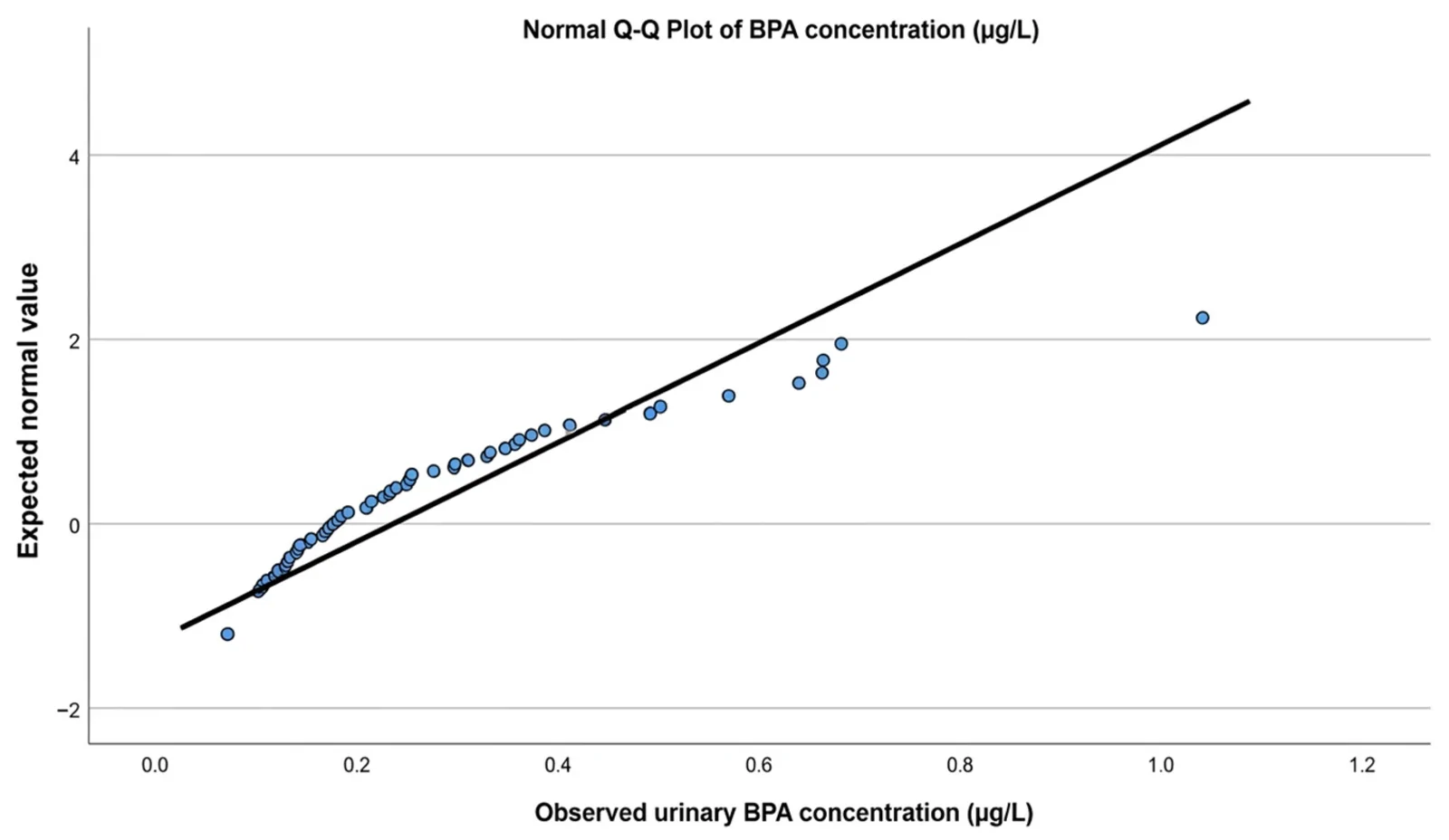
Expected normal values of BPA in urine samples of pregnant women based on observed values. Normal values of BPA were expected at concentrations up to 0.400 μg/L. In a proportion of samples (10%—8/77 samples), BPA concentrations exceeded these values, even 1.000 μg/L, and were expected to be abnormal.

**Figure 3:**
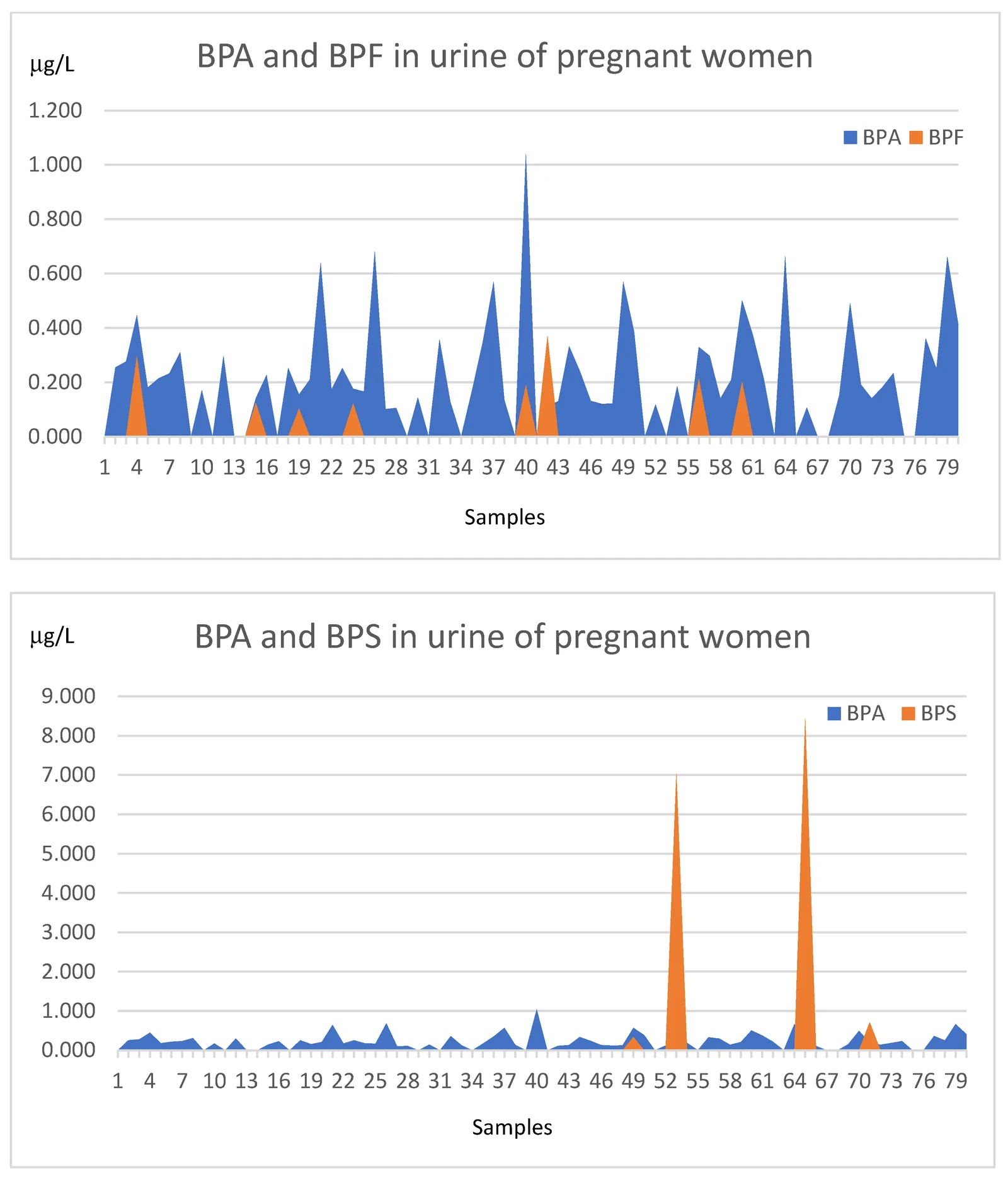
Concentrations of bisphenols—BPA, BPF, and BPS (μg/L)—above LODs in urine samples of pregnant women. BPA was the most prominent EDC in urine samples, detected in 78%, while BPF and BPS were detected in a lower proportion (10% and 5%, respectively).

**Figure 4:**
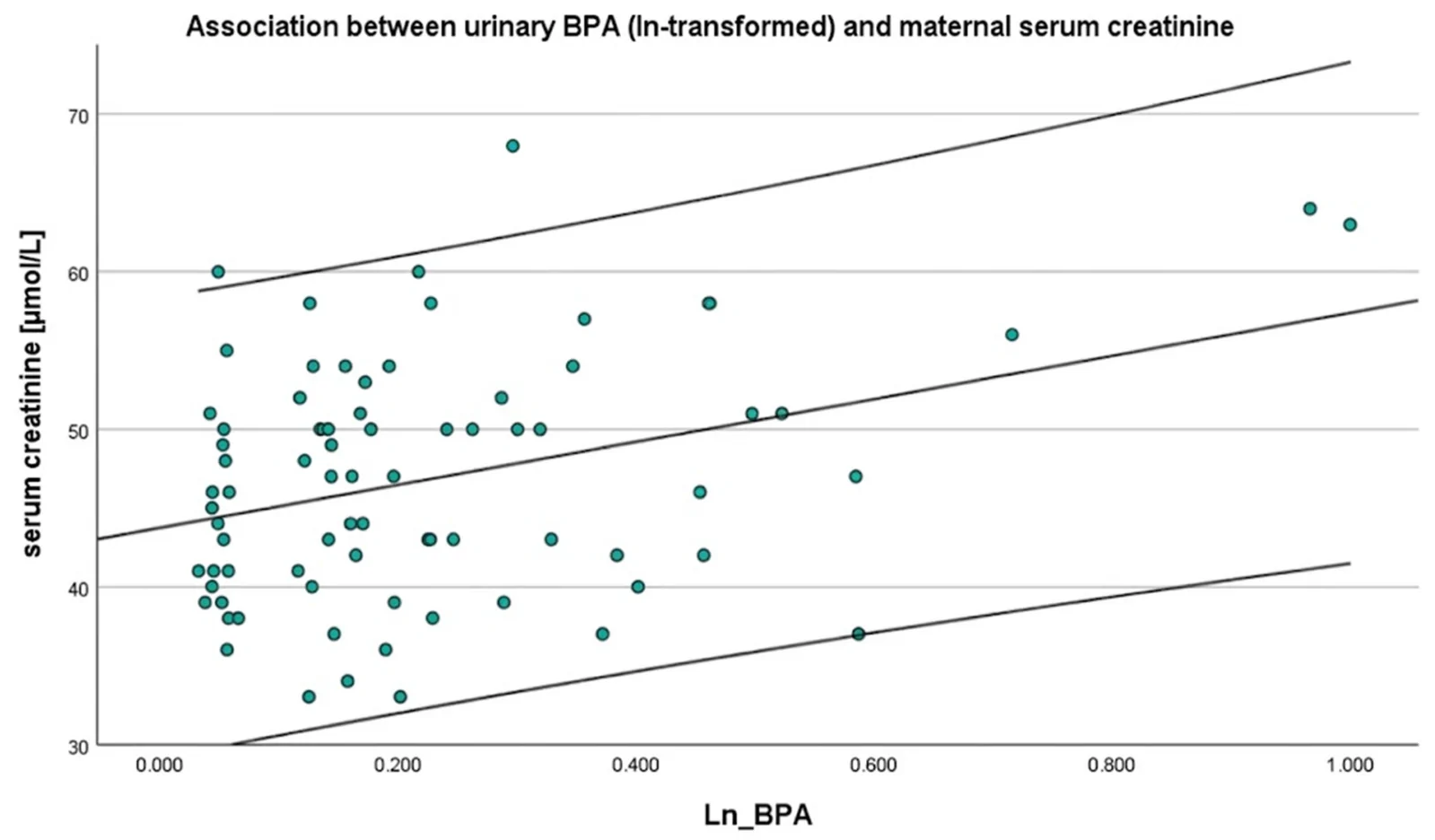
Positive association between ln-transformed urinary BPA concentrations and maternal serum creatinine in the full cohort. The middle line represents the fitted regression line, with the outer lines indicating 95% confidence intervals.

**Table 1:** Baseline characteristics of the study population of pregnant women.

Characteristics of the Study Population		
Body mass index [kg/m^2^] median (min–max)		24.05 (18.2–40.7)
Age [years] median (min–max)		33.00 (23–39)
Parity median (min–max)		2.00 (1–4)
Gestational age at delivery and sampling [weeks] median (min–max)		39 (28–41)
Residence in the Central Slovenian region *n* (%)	No	18 (22.5)
	Yes	62 (77.5)
Spontaneous conception *n* (%)	No	4 (5)
	Yes	76 (95)
Gestational diabetes mellitus (diet-controlled) *n* (%)	No	57 (71.3)
	Yes	23 (28.7)
Smoking status *n* (%)	non smoker	58 (96.7)
	Smoker	2 (3.3)
Education *n* (%)	Secondary education	25 (42.4)
	Tertiary education	34 (57.6)
Serum creatinine [µmol/L] mean ± SD		46.78 ± 7.66
eGFR [mL/min/1.73 m^2^] median (min–max)		147.8 (141.2–154.4)

Legend: eGFR—estimated glomerular filtration rate; SD—standard deviation; min—minimum; max—maximum; *n*—count; %—percentage.

**Table 2:** Neonatal anthropometric and clinical characteristics at birth.

Neonatal Characteristics		
Gestational age at delivery [weeks] median (min–max)		39 (28–41)
Newborn sex *n* (%)	Female	40 (50)
	Male	40 (50)
Birth weight [g] median (min–max)		3520 (820–4850)
Birth length [cm] median (min–max)		51 (35–59)
Head circumference [cm] median (min–max)		35 (26–39)
APGAR [1 min] median (min–max)		9 (8–10)
APGAR [5 min] median (min–max)		9 (9–10)
pH (A) median (min–max)		7.30 (7.02–7.37)
pH (V) median (min–max)		7.35 (7.09–7.42)

Legend: APGAR (1 min), Apgar score at 1 min; APGAR (5 min), Apgar score at 5 min; pH (A), umbilical arterial blood pH; pH (V), umbilical venous blood pH; min, minimum; max, maximum; *n*, count; %, percentage.

**Table 3:** Correlation analysis of urinary BPA association with female characteristics. There was no significant association (Spearman correlation, statistical significance at *p* < 0.05).

Female Characteristics	Region of Residence	Way of Conception	BMI	Parity	No. of Previous Pregnancies	Spontaneous Abortions	GDM	Hypothyroidism
*p*-value	0.993	0.9276	0.3732	0.1547	0.4111	0.1547	0.7312	0.7949

Legend: BMI—body mass index; GDM—gestational diabetes mellitus.

**Table 4:** Correlations between BPA in maternal urine and neonatal characteristics. There was a significant negative correlation with neonatal weight and head circumference.

Delivery Characteristics	Gestation (Weeks)	Newborn Weight	Newborn Length	Newborn Head Circumference	Newborn Gender	Apgar (1 min)	pH Arterial	pH Venous
*p*-value	0.0072 *	0.046 *	0.106	0.022 *	0.667	0.4119	0.7597	0.1779

* statistically significant (Spearman correlation, *p* < 0.05).

**Table 5:** Correlations of BPA with urine and biochemical parameters related to kidney and liver function, AMH, and vitamin D3.

Kidney and Liver Function	Urine	Serum									
	SG	CRC	AST	ALT	CHO	γ-GT	HDL	LDL	TG	AMH	VIT. D3
*p*-value	<0.001 *	0.762	0.723	0.848	0.262	0.075	0.927	0.543	0.004 *	0.049 *	0.592

* statistically significant (Spearman correlation, *p* < 0.05). Legend: SG—specific gravity; CRC—creatinine; CHO—cholesterol; TG—triglycerides; VIT. D3—vitamin D3.

**Table 6:** Multivariable linear regression analysis of the association between urinary BPA concentrations adjusted for urine specific gravity (SG) or creatinine (CRC) and maternal serum creatinine in pregnant women. The table shows a consistent significant association between urinary BPA and serum creatinine in these women.

Dependent Variable	Multivariable Linear Regression Model	B Value	95.0% Confidence Interval for B		*p*-Value
			Lower Bound	Upper Bound	
Serum creatinine	primary (LnBPA, BMI, maternal age, gestation) with SG	14.913	4.414	25.411	0.006 *
	primary with SG and GDM	13.419	3.036	23.801	0.012 *
	primary with SG and parity	14.771	4.142	25.401	0.007 *
	primary with SG and education	14.514	3.783	25.246	0.009 *
	primary (LnBPA, BMI, maternal age, gestation) with CRC	15.260	4.426	26.094	0.007 *
	primary with CRC and GDM	14.208	3.556	24.86	0.01 *
	primary with CRC and parity	15.163	4.189	26.138	0.008 *
	primary with CRC and education	14.820	3.795	25.844	0.01 *

* statistically significant (multivariable linear regression, *p* < 0.05). Legend: B value—regression coefficient; lnBPA—ln-transformed urinary BPA concentrations; GDM—gestational diabetes mellitus; SG—specific gravity; CRC—creatinine.

**Table 7:** Multivariable linear regression analysis of the association between maternal BPA in urine adjusted for urine specific gravity and outcomes in female neonates and maternal biochemical parameters.

**Neonatal Outcomes for Female Newborns**					
**Parameters**	**MODEL**	**B Value**	***p*** **-Value**	**95.0% Confidence Interval for B**	
	**Results for BPA**			**Lower Bound**	**Upper Bound**
Gestational age at birth	primary (LnBPA, BMI, maternal age, education) with SG *	2.64	0.266	−2.169	7.45
Birth weight	primary (LnBPA, BMI, maternal age, gestational age) with SG *	159.009	0.57	−412.46	730.48
Birth length	primary (LnBPA, BMI, maternal age, gestational age) with SG *	−1.383	0.389	−4.641	1.874
Head circumference	primary (LnBPA, BMI, maternal age, gestational age) with SG *	0.011	0.991	−2.111	2.134
APGAR1	primary (LnBPA, BMI, maternal age, gestational age) with SG *	−0.048	0.87	−0.648	0.552
pHa	primary (LnBPA, BMI, maternal age, gestational age) with SG *	0.063	0.207	−0.037	0.163
pHv	primary (LnBPA, BMI, maternal age, gestational age) with SG *	0.09	0.099	−0.018	0.199
**Maternal Biochemistry Parameters**					
**Parameters**	**MODEL**	**B**	***p*** **-Value**	**95.0% Confidence Interval for B**	
	**Results for BPA**			**Lower Bound**	**Upper Bound**
AMH	primary (LnBPA, BMI, maternal age, gestation) with SG *	−0.601	0.484	−2.393	1.191
Serum creatinine	primary (LnBPA, BMI, maternal age, gestation) with SG *	13.489	0.105	−3.063	30.041
S-AST	primary (LnBPA, BMI, maternal age, gestation) with SG *	0.133	0.3	−0.127	0.394
S-ALT	primary (LnBPA, BMI, maternal age, gestation) with SG *	−0.044	0.7	−0.276	0.188
S-gamma-GT	primary (LnBPA, BMI, maternal age, gestation) with SG *	−0.012	0.866	−0.161	0.137
S-cholesterol	primary (LnBPA, BMI, maternal age, gestation) with SG *	1.559	0.361	−1.904	5.021
S-HDL cholesterol	primary (LnBPA, BMI, maternal age, gestation) with SG *	0.156	0.759	−0.882	1.194
S-LDL cholesterol	primary (LnBPA, BMI, maternal age, gestation) with SG *	−3.378	0.137	−7.923	1.166
S-TG	primary (LnBPA, BMI, maternal age, gestation) with SG *	−1.125	0.342	−3.524	1.274
S-leukocytes	primary (LnBPA, BMI, maternal age, gestation) with SG *	2.044	0.433	−3.254	7.341
S-erythrocytes	primary (LnBPA, BMI, maternal age, gestation) with SG *	−0.538	0.232	−1.445	0.369
Hb	primary (LnBPA, BMI, maternal age, gestation) with SG *	−9.845	0.482	−38.348	18.659
Ht	primary (LnBPA, BMI, maternal age, gestation) with SG *	−0.036	0.368	−0.118	0.045
MCV	primary (LnBPA, BMI, maternal age, gestation) with SG *	2.672	0.622	−8.398	13.742
MCH	primary (LnBPA, BMI, maternal age, gestation) with SG *	1.324	0.572	−3.45	6.099
MCHC	primary (LnBPA, BMI, maternal age, gestation) with SG *	7.883	0.501	−15.971	31.737
RDW	primary (LnBPA, BMI, maternal age, gestation) with SG *	0.416	0.842	−3.856	4.688
Platelets	primary (LnBPA, BMI, maternal age, gestation) with SG *	105.098	0.02	18.006	192.189
MPV	primary (LnBPA, BMI, maternal age, gestation) with SG *	−1.357	0.3	−4.001	1.288
Urinary IgG	primary (LnBPA, BMI, maternal age, gestation) with SG *	0.517	0.959	−20.061	21.095
Urinary albumin	primary (LnBPA, BMI, maternal age, gestation) with SG *	−1.678	0.974	−107.764	104.407
Urinary alpha1 microglobulin	primary (LnBPA, BMI, maternal age, gestation) with SG *	−10.65	0.533	−45.746	24.447
Urinary IgG/albumin ratio	primary (LnBPA, BMI, maternal age, gestation) with SG *	−0.278	0.605	−1.573	1.017
Urinary N-acetyl-β-D-glucosaminidase (NAG)	primary (LnBPA, BMI, maternal age, gestation) with SG *	148.685	0.501	−303.021	600.391
Serum 25-hydroxyvitamin D	primary (LnBPA, BMI, maternal age, gestation) with SG *	4.019	0.885	−53.155	61.192

* adjusted for urine specific gravity. Legend: B—unstandardized regression coefficient; BMI—body mass index; AMH—anti-Müllerian hormone; AST—aspartate aminotransferase; ALT—alanine aminotransferase; HDL—high-density lipoprotein cholesterol; LDL—low-density lipoprotein cholesterol; Hb—hemoglobin; Ht—hematocrit; MCV—mean corpuscular volume; MCH—mean corpuscular hemoglobin; MCHC—mean corpuscular hemoglobin concentration; RDW—red cell distribution width; MPV—mean platelet volume; NAG—N-acetyl-β-D-glucosaminidase; LnBPA—natural logarithm of urinary BPA concentration; SG—urine specific gravity.

**Table 8:** Multivariable linear regression analysis of the association between BPA adjusted for urine specific gravity and outcomes in male neonates and maternal biochemical parameters.

**Neonatal Outcomes for Male Newborns**					
**Parameters**	**MODEL**	**B Value**	***p*** **-Value**	**95.0% Confidence Interval for B**	
	**Results for LnBPA**			**Lower Bound**	**Upper Bound**
Gestational age at birth	primary (LnBPA, BMI, education, maternal age) with SG *	−1.574	0.62	−8.032	4.885
Birth weight	primary (LnBPA, BMI, maternal age, gestational age) with SG *	19.89	0.967	−952.15	991.93
Birth length	primary (LnBPA, BMI, maternal age, gestational age) with SG *	−1.286	0.571	−5.913	3.34
Head circumference	primary (LnBPA, BMI, maternal age, gestational age) with SG *	0.554	0.721	−2.609	3.717
APGAR1	primary (LnBPA, BMI, maternal age, gestational age) with SG *	−0.231	0.172	−0.569	0.108
pHa	primary (LnBPA, BMI, maternal age, gestational age) with SG *	0.013	0.807	−0.095	0.12
pHv	primary (LnBPA, BMI, maternal age, gestational age) with SG *	0.015	0.846	−0.145	0.175
**Maternal Biochemistry Parameters**					
**Parameters**	**MODEL**	**B**	***p*** **-Value**	**95.0% Confidence Interval for B**	
	**Results for LnBPA**			**Lower Bound**	**Upper Bound**
AMH	primary (LnBPA, BMI, maternal age, gestation) with SG *	2.316	0.097	−0.479	5.111
Serum creatinine	primary (LnBPA, BMI, maternal age, gestation) with SG *	21.126	0.014	4.621	37.632
S-AST	primary (LnBPA, BMI, maternal age, gestation) with SG *	0.118	0.467	−0.211	0.447
S-ALT	primary (LnBPA, BMI, maternal age, gestation) with SG *	−0.105	0.414	−0.367	0.156
S-gamma-GT	primary (LnBPA, BMI, maternal age, gestation) with SG *	0.128	0.093	−0.023	0.279
S-cholesterol	primary (LnBPA, BMI, maternal age, gestation) with SG *	−0.36	0.796	−3.208	2.488
S-HDL cholesterol	primary (LnBPA, BMI, maternal age, gestation) with SG *	−0.085	0.86	−1.065	0.896
S-LDL cholesterol	primary (LnBPA, BMI, maternal age, gestation) with SG *	−0.029	0.979	−2.321	2.262
S-TG	primary (LnBPA, BMI, maternal age, gestation) with SG *	0.903	0.248	−0.673	2.479
S-leukocytes	primary (LnBPA, BMI, maternal age, gestation) with SG *	2.543	0.197	−1.415	6.501
S-erythrocytes	primary (LnBPA, BMI, maternal age, gestation) with SG *	−0.263	0.389	−0.882	0.356
Hb	primary (LnBPA, BMI, maternal age, gestation) with SG *	−0.461	0.964	−21.611	20.688
Ht	primary (LnBPA, BMI, maternal age, gestation) with SG *	−0.001	0.969	−0.068	0.066
MCV	primary (LnBPA, BMI, maternal age, gestation) with SG *	5.603	0.237	−3.935	15.141
MCH	primary (LnBPA, BMI, maternal age, gestation) with SG *	2.023	0.183	−1.028	5.074
MCHC	primary (LnBPA, BMI, maternal age, gestation) with SG *	−12.818	0.167	−31.407	5.771
RDW	primary (LnBPA, BMI, maternal age, gestation) with SG *	−1.208	0.501	−4.867	2.451
Platelets	primary (LnBPA, BMI, maternal age, gestation) with SG *	−21.313	0.666	−122.11	79.484
MPV	primary (LnBPA, BMI, maternal age, gestation) with SG *	2.993	0.034	0.252	5.735
Urinary IgG	primary (LnBPA, BMI, maternal age, gestation) with SG *	−58.046	0.461	−219.813	103.72
Urinary albumin	primary (LnBPA, BMI, maternal age, gestation) with SG *	−459.646	0.448	−704.018	784.726
Urinary alpha1 microglobulin	primary (LnBPA, BMI, maternal age, gestation) with SG *	−5.198	0.662	−29.974	19.579
Urinary IgG/albumin ratio	primary (LnBPA, BMI, maternal age, gestation) with SG *	0.655	0.395	−1.968	3.278
Urinary N-acetyl-β-D-glucosaminidase (NAG)	primary (LnBPA, BMI, maternal age, gestation) with SG *	17.098	0.843	−159.585	193.782
Serum 25-hydroxyvitamin D	primary (LnBPA, BMI, maternal age, gestation) with SG *	80.707	0.116	−21.443	182.856

* adjusted for urine specific gravity. Legend: B—unstandardized regression coefficient; BMI—body mass index; AMH—anti-Müllerian hormoneAST—aspartate aminotransferase; ALT—alanine aminotransferase; HDL—high-density lipoprotein cholesterol; LDL—low-density lipoprotein cholesterol; Hb—hemoglobin; Ht—hematocrit; MCV—mean corpuscular volume; MCH—mean corpuscular hemoglobin; MCHC—mean corpuscular hemoglobin concentration; RDW—red cell distribution width; MPV—mean platelet volume; NAG—N-acetyl-β-D-glucosaminidase; LnBPA—natural logarithm of urinary BPA concentration; SG—urine specific gravity.

## Data Availability

The original contributions presented in this study are included in the article/Supplementary Material. Further inquiries can be directed to the corresponding authors.

## Supplementary Materials

The following supporting information can be downloaded at: https://www.mdpi.com/article/10.3390/jox16060180/s1, Table S1. Maternal demographic, obstetric, and biochemical characteristics of the study population. Table S2. Neonatal anthropometric and clinical characteristics at birth. Table S3. Maternal demographic and obstetric characteristics of the study population (categorical variables). Table S4. Geometric means and geometric standard deviations of urinary bisphenol concentrations. Table S5. Concentrations of BPA in urine of pregnant women in this and other studies worldwide.

## Abbreviations

The following abbreviations are used in this manuscript:

ALT	Alanine transaminase
AMH	Anti-Müllerian hormone
AST	Aspartate aminotransferase
BMI	Body mass index
BPA	Bisphenol A
BPF	Bisphenol F
BPS	Bisphenol S
CHO	Cholesterol
CRC	Creatinine
EDC	Endocrine-disrupting chemical
eGFR	Estimated glomelural filtration rate
GDM	Gestational diabetes mellitus
Hb	Hemoglobin
HDL	High-density lipoprotein cholesterol
Ht	Hematocryt
LDL	Low-density lipoprotein cholesterol
LOD	Limit of detection
MCH	Mean erythrocyte hemoglobin content
MCHC	Mean erythrocyte concentration of hemoglobin
MCV	Mean erythrocyte volume
MPV	Mean platelet volume
NAG	N-acetyl-β-d-glucosaminidase
PLT	Platelet count
RBC	Red blood cell count
RDW	Volume distribution of erythrocytes
SD	Standard deviation
SG	Specific gravity
TG	Triglycerides
VIT.D3	Vitamin D3
WBC	White blood cell count
