# Supplementary material for: Potential Adverse Effects of Urinary Bisphenol A Concentrations on Renal Function, Gestation, and Newborn Characteristics in Healthy Pregnant Women
Source: J Xenobiot. 2026 Sep 24;16(6):180. doi: 10.3390/jox16060180 (PMC13706542; doi:10.3390/jox16060180)
Supplement: Supplementary file 1 [file jox-16-00180-s001.zip › jox-4496728-supplementary.pdf]

Supplementary Materials: Potential Adverse Effects of Urinary Bisphenol A Concentrations on Renal Function, Gestation, and Newborn Characteristics in Healthy Pregnant Women

Senka Imamović Kumalić, Gorazd Kavšek, Mateja Sladič, Tanja Premru Sršen and Irma Virant-Klun

Table S1. Maternal demographic, obstetric, and biochemical characteristics of the study population.

Parameters	Number		Mean	Standard Deviation	Median	Minimum	Maximum	Range
	Valid	Missing						
Body mass index (kg/m²)	80	0	25.71	5.17	24.05	18.2	40.7	22.5
Age (years)	60	20	32.13	4.20	33.00	23	39	16
Number of pregnancies	80	0	2.48	1.13	2.00	1	6	5
Parity	80	0	1.95	0.69	2.00	1	4	3
Number of miscarriages	80	0	0.39	0.74	0.00	0	3	3
Gestational age at delivery and sampling (weeks)	80	0	38.81	2.29	39.00	28	41	13
AMH (S-A1) (ng/L)	60	20	1.02	0.87	0.85	0.17	5.5	5.33
urine specific gravity	79	1	1.02	0.02	1.01	1.004	1.21	0.206
serum creatinine (µmol/L)	77	3	46.78	7.66	47.00	33	68	35
eGFR (mL/min/1.73 m²)	78	2	95.00	0.00	95.00	95	95	0
S-AST (µkat/L)	79	1	0.41	0.13	0.38	0.18	0.97	0.79
S-ALT (µkat/L)	79	1	0.25	0.11	0.22	0.05	0.63	0.58
S-gama-GT (µkat/L)	79	1	0.10	0.07	0.08	0	0.31	0.31
Serum cholesterol (mmol/L)	80	0	7.02	1.40	6.90	4.6	11.5	6.9
S-HDL-Holesterol (mmol/L)	80	0	1.94	0.45	1.95	1.1	3.3	2.2
S-LDL (mmol/L)	78	2	4.07	1.07	3.95	2.1	6.3	4.2
serum triglycerides (mmol/L)	80	0	2.87	1.04	2.80	1.2	6.6	5.4
blood leukocytes (WBC) ($\times 10^9$/L)	79	1	10.59	2.40	10.10	7.2	19	11.8
blood erythrocytes (RBC) ($\times 10^{12}$/L)	79	1	4.09	0.35	4.09	3.16	5.2	2.04
K-Hb (g/L)	79	1	120.42	10.68	121.00	99	149	50
K-Ht (L/L)	79	1	0.36	0.03	0.36	0.3	0.442	0.142
MCV (fL)	79	1	87.86	5.31	88.10	71.6	99.3	27.7
MCH (pg)	79	1	29.47	1.89	29.50	23.4	33.5	10.1
MCHC (g/L)	79	1	336.22	9.06	337.00	312	356	44
RDW (%)	79	1	15.18	1.78	14.50	13.1	19.9	6.8
platelets (PLT) ($\times 10^9$/L)	79	1	216.23	62.70	210.00	107	458	351
MPV (fL)	79	1	11.06	1.24	11.00	8.4	14.2	5.8
urinary IgG/creatinine ratio (g/mol)	66	14	1.21	6.00	0.01	0.01	48.77	48.76
urinary_IgG	67	13	10.17	43.87	0.10	0	356.02	356.02
urinary albumin/creatinine ratio (g/mol)	67	13	7.91	45.34	1.47	0.01	372.6	372.59
urinary_albumin	66	14	64.68	335.06	8.23	0.02	2719.98	2719.96

urinary α1-microglobulin/creatinine ratio (g/mol)	64	16	0.96	1.03	0.79	0.01	3.9	3.89
urinary_alpha1microglobulin	64	16	9.08	15.76	6.83	0.02	114.87	114.85
urinary IgG/albumin ratio	28	52	0.36	0.22	0.28	0.08	0.89	0.81
urine creatinine	78	2	6.96	5.80	5.10	0.5	34.6	34.1
urinary NAG/creatinine ratio (µkat/mol of creatinine)	64	16	20.43	11.28	18.89	6.15	53.04	46.89
urinary N-acetyl-β-D-glucosaminidase (NAG)	75	5	146.77	213.64	95.90	12.8	1603.60	1590.80
serum 25-hydroxyvitamin D (nmol/L)	75	5	64.91	39.19	59.00	17	214	197
BPA (µg/L)	60	20	0.28	0.19	0.22	0.101	1.041	0.939
BPF (µg/L)	8	72	0.20	0.09	0.19	0.102	0.369	0.267
BPS (µg/L)	4	76	4.13	4.21	3.87	0.334	8.439	8.105
Imp_BPA (µg/L)	77	3	0.23	0.19	0.18	0.036	1.041	1.005
BPA_SG (µg/L)	77	3	0.22	0.15	0.18	0.01	0.96	0.95
BPA_Crc (µg/g of creatinine)	75	5	0.39	0.30	0.32	0.04	1.73	1.69

Legend: BMI – body mass index; d-ROM – derivatives of reactive oxygen metabolites; AMH – anti-Müllerian hormone; eGFR – estimated glomerular filtration rate; AST – aspartate aminotransferase; ALT – alanine aminotransferase; γ-GT – gamma-glutamyl transferase; HDL – high-density lipoprotein cholesterol; LDL – low-density lipoprotein cholesterol; WBC – white blood cell count; RBC – red blood cell count; Hb – hemoglobin; Ht – hematocrit; MCV – mean corpuscular volume; MCH – mean corpuscular hemoglobin; MCHC – mean corpuscular hemoglobin concentration; RDW – red cell distribution width; PLT – platelet count; MPV – mean platelet volume; NAG – N-acetyl-β-D-glucosaminidase; BPA – bisphenol A; BPF – bisphenol F; BPS – bisphenol S.

Table S2. Neonatal anthropometric and clinical characteristics at birth.

**Parameters**	**Number**		**Mean**	**Standard Deviation**	**Median**	**Minimum**	**Maximum**	**Range**
	**Valid**	**Missing**						
Gestational age at delivery (weeks)	80	0	38.81	2.29	39.00	28	41	13
Birth weight (g)	80	0	3369	681	3520	820	4850	4030
Birth length (cm)	80	0	50.53	3.65	51.00	35	59	24
Head circumference (cm)	80	0	34.76	2.23	35.00	26	39	13
APGR (1.min)	80	0	9.01	0.25	9.00	8	10	2
APGAR (5. min)	80	0	9.05	0.22	9.00	9	10	1
pH (A)	74	6	7.29	0.05	7.30	7.02	7.37	0.35
pH (V)	72	8	7.34	0.05	7.35	7.09	7.42	0.33

Legend**:** APGAR (1 min) – Apgar score at 1 minute; APGAR (5 min) – Apgar score at 5 minutes; pH (A), umbilical arterial blood pH; pH (V), umbilical venous blood pH.

Table S3. Maternal demographic and obstetric characteristics of the study population (categorical variables).

**Parameters**	**Categories**	**Frequency**	**Percent**
Residence in the Central Slovenia Region	No	18	22.5
	Yes	62	77.5
Spontaneous conception	No	4	5
	Yes	76	95
Gestational diabetes mellitus (diet-controlled)	No	57	71.3
	Yes	23	28.7
Hypothyroidism	No	76	95
	Yes	4	5
Newborn gender	female	40	50
	male	40	50

Meconium	No	75	93.8
	Yes	5	6.3
Smoking status	non smoker	58	96.7
	Yes	2	3.3
Education	Lower secondary education	21	35.6
	Upper secondary education	4	6.8
	Tertiary education	18	30.5
	Bachelor's degree	16	27.1
Education: secondary vs tertiary	Secondary education	25	42.4
	Tertiary education	34	57.6

Table S4. Geometric means and geometric standard deviations of urinary bisphenol concentrations

Bisphenols	Geometric mean	Geometric standard deviation
IMP_BPA_SG	0.173	2.103
IMP_BPA_Crc	0.303	2.150
IMP_BPA	1.248	1.212
BPA	1.323	1.208
BPF	1.337	1.328
BPS	4.380	3.518

Table S5. Concentrations of BPA in urine of pregnant women in this and other studies worldwide.

	BPA concentrations in urine of pregnant women		
**Study (country)**	**Detection rate**	**Geometric mean (SD)**	**Median**
Our study (Slovenia)	78 % (n = 77)	0.2373 µg/L (0.1841) 0.3529 µg/g creatinine (0.1184)	0.22 µg/L 0.3526 µg/g creatinine (third trimester)
Li et al., 2024 (China) [37]	100 % (n = 113)		5.84 µg/L (third trimester)
Suwannarin et al., 2024 (Japan) [38]	71.5 % (n = 4577)		0.46 µg/g creatinine
Chen et al., 2022 (USA, Oklahoma) [39]	93.3 % (n = 314)		2.58 (2.29, 2.91) ng/mL (SG-adjusted) (mid-pregnancy, median: 26.0 gestational weeks)
Forde et al., 2022 (10 Carribean islands) [40]	100 % (n = 150)	from 1.46 µg/L (St. Lucia) to 4.88 µg/L (St. Kitts & Nevis)	
Kim et al., 2021 (USA, California, y. 2007-2014) [41]	59 % (n = 218)		1.0 ng/mL. (SG-adjusted)
Martinez et al., 2021 (Spain) [42]	76 % (n = 60)		4.64 µg/g creatinine (first trimester) 4.84 µg/g creatinine (second trimester) 2.51 µg/g creatinine (third trimester)
Guo et al., 2020 (China) [43]	/ (n = 386)		1.75 µg/L
Mehlsen et al., 2022 (Denmark) [44]	/ (n = 725)		1.29 ng/L (third trimester)
Ashley-Martin et al., 2021 (Canada) [45]	/ (n = 1879)		0.81 µg/L (first trimester)

Betul et al., 2022 (Turkey) [46]	/ (n = 30)	2.55 μg/g creatinine (first trimester) 2.27 μg/g creatinine (second trimester) 1.58 μg/g creatinine (third trimester)
